# Transcriptome Analysis of Breast Muscle Reveals Pathways Related to Protein Deposition in High Feed Efficiency of Native Turkeys

**DOI:** 10.3390/ani12101240

**Published:** 2022-05-11

**Authors:** Zahra Pezeshkian, Seyed Ziaeddin Mirhoseini, Shahrokh Ghovvati, Esmaeil Ebrahimie

**Affiliations:** 1 Department of Animal Sciences, Faculty of Agriculture, University of Guilan, Rasht 41635-1314, Guilan, Iran; zpezeshkian@webmail.guilan.ac.ir (Z.P.); Ghovvati@guilan.ac.ir (S.G.); 2 Genomics Research Platform, School of Agriculture, Biomedicine and Environment, La Trobe University, Melbourne, VIC 3086, Australia; 3 School of Animal and Veterinary Sciences, The University of Adelaide, Adelaide, SA 5371, Australia; 4 School of BioSciences, The University of Melbourne, Melbourne, VIC 3010, Australia

**Keywords:** feed efficiency, Turkey, conversion coefficient, RNA-Seq, different gene expression analysis

## Abstract

**Simple Summary:**

Due to the high cost of feed in the poultry production system, improving feed efficiency can reduce production costs. This study aimed to investigate the transcriptome of breast muscle tissue of native male turkeys that have been phenotyped for high and low feed efficiency. After the rearing period, the most efficient and the least efficient male turkeys were selected and slaughtered for RNA-Seq. Genes with different expressions in muscle tissue were identified between high feed efficiency turkeys compared to low feed efficiency turkeys. The results showed that high feed efficiency birds increased the expression of genes related to amino acid biosynthesis and low feed efficiency birds increased the expression of genes related to the immune response. Eleven key genes associated with the phenotypic expression of feed efficiency were selected that may be good potential candidates for biomarkers of feed efficiency in genetic selection in turkeys. This study provides new insights into the differences in transcripts between breast muscle tissues between turkeys with high and low feeding efficiencies.

**Abstract:**

Feed efficiency is important due to the high cost of food, which accounts for about 70% of the total cost of a turkey breeding system. Native poultry are an important genetic resource in poultry breeding programs. This study aimed to conduct a global transcriptome analysis of native male turkeys which have been phenotyped for high and low feed efficiency. Feed efficiency traits were recorded during the experimental period. After slaughter, the three most efficient and three least efficient male turkeys were selected for RNA-Seq analysis. A total of 365 genes with different expressions in muscle tissue were identified between turkeys with a high feed efficiency compared to turkeys with a low feed efficiency. In the pathway analysis of up-regulated genes, major pathways included the “metabolism of glycine, serine, and threonine”; the “adipocytokine signaling pathway” and the “biosynthesis of amino acids”. In the pathway analysis of down-regulated genes, the major pathways included “dorso-ventral axis formation” and “actin cytoskeleton regulation”. In addition, gene set enrichment analyses were performed, which showed that high feed efficiency birds exhibit an increased expression of genes related to the biosynthesis of amino acids and low feed efficiency birds an increased expression of genes related to the immune response. Furthermore, functional analysis and protein network interaction analysis revealed that genes including *GATM*, *PSAT1*, *PSPH*, *PHGDH*, *VCAM1*, *CD44*, *KRAS*, *SRC*, *CAV3*, *NEDD9,* and *PTPRQ* were key genes for feed efficiency. These key genes may be good potential candidates for biomarkers of feed efficiency in genetic selection in turkeys.

## 1. Introduction

Feed cost is an important component of poultry production which accounts for 70% of the total production costs [1,2]. Therefore, feed efficiency (FE) is one of the most important issues in poultry breeding programs. Improving feed efficiency may decrease feed intake, production cost, waste products, and environmental emissions [3,4]. As a result of genetic progress, larger birds need more feed. By improving feed efficiency, birds that eat the same amount of feed as other birds have higher body weight or weight gain [1]. The turkey is an important agricultural species used for meat production. Global data from the turkey industry show that turkey consumption has increased in recent years [4]. Willems et al. (2013) evaluated different feed efficiency measurements in turkeys [2].

Understanding the fundamental basis for a bird to have the optimal ability to convert feed into high-quality meat protein is important to feed the human population and to maintain healthy and sustainable animals in the future [5].

Many biochemical and functional differences related to the expression of feed efficiency exist within a single line of poultry [6]. Insights into the biological basis of differences in poultry feed efficiency are required to develop more efficient and sustainable selection strategies. With the rapid development of next-generation sequencing technologies, RNA sequencing (RNA-Seq) has been replacing microarray technology for transcriptome-wide gene expression analysis. Avoiding the technical issues inherent to the microarray technique such as cross-hybridization and narrow ranges of signal detection, RNA-Seq can provide more accurate and comprehensive information regarding changes in gene expression between different conditions or different phenotypes [7].

Many studies have examined the global gene expression of feed efficiency in different animals such as chickens using microarray and RNA-Seq technology [5,7,8,9,10,11]. Moreover, the RNA-Seq method has attracted considerable interest and achieved great success concerning many economic traits in livestock [11,12]. An RNA-Seq analysis of abdominal fat revealed differences between high and low commercial broiler chickens. One of the important pathways implicated was the pathway of amino acid metabolism [13]. The key genes and signaling pathways associated with feed efficiency in native chickens have been studied, and it has been reported that the metabolic pathways are significantly different in one of the modules of the gene network [14].

Therefore, a global gene expression study using RNA-Seq is necessary for a better understanding of the molecular basis of FE in turkeys. However, to our knowledge, there are no reports on global gene expression associated with feed efficiency in turkeys. This study aimed to conduct a transcriptome analysis of domestic turkeys that were individually phenotyped for feed efficiency. Our findings will allow a better understanding of the underlying mechanisms of feed efficiency and help to optimize the current breeding strategies.

## 2. Materials and Methods

### 2.1. Ethical Approval Statements

This research was conducted under the ethical standards of the University of Guilan. All animal work was approved by Vice Presidency for Research and Technology of the University of Guilan (Number: 25433/p15). Before sampling, birds were humanely slaughtered by cervical dislocation. Every effort was made to minimize their suffering.

### 2.2. Selecting Animals and Sampling

In this research, Iranian native turkeys of the Tatar Turkey Research Center were used. Five hundred male turkeys were raised until 20 weeks of age under a standard production protocol, which included a standard diet and housing in groups with common drinkers and feeders. Out of this group of 500 turkeys, 75 were randomly selected and moved to the farm of the University of Guilan. They were placed into individual cages from week 20 to 24 of age with feed and water ad libitum. A lighting regimen of 16 h of light and 8 h of darkness was used.

Body weight was evaluated at the beginning (20 weeks of age) and the end of the experiment (24 weeks of age). Feed consumption was recorded by measuring the weight of the food added to each feeder daily and weighing the residual feed weekly until the end of the experiment. Feed conversion ratio (FCR) was computed as:

(1) $$\mathrm{FCR}=\frac{\mathrm{FI}\left(g\right)}{\mathrm{WG}\;\left(g\right)}$$

where FI is the total feed intake during the trial and WG is the body weight gain during this time.

Turkeys were ranked by FCR and the 3 lowest and 3 highest FCR-ranked toms were assigned as high feed efficiency (HFE) and low feed efficiency (LFE), respectively, and used for RNA-Seq. After slaughter, breast muscle tissues were harvested immediately, frozen in liquid nitrogen, and then stored at −80 °C until further processing.

### 2.3. RNA Extraction and Sequencing

The total RNA was isolated using the RNeasy Midi kit (Qiagen, Hilden, Germany, Cat. # 75144). The RNA quantity was evaluated by measuring the absorbance at 260, 280, and 230 nm using a spectrophotometer (EPOCH 2, Biotek Instruments Inc., Winooski, VT, USA). The reference 260/280 ratio and 260/230 ratio for the RNA sample were 1.8 to 2.0 and 1.8 to 2.2, respectively. The quality of RNA samples was checked using 1% agarose gels. The RNA integrity number (RIN) was accessed using a Agilent Bioanalyzer 2100 system (Agilent Technologies, Santa Clara, CA, USA). Only RNA integrity numbers equal to or higher than 7.0 were used for the next analysis. RNA library preparation and Illumina sequencing were performed by Novogene Biotechnology Co. Ltd. (Beijing, China). The library was prepared with TruSeq RNA Sample Prep Kit v2 (Illumina, San Diego, CA, USA). Illumina HiSeq 2500 system technology was applied for RNA sequencing, and the length of the paired-end short reads was 150 bp.

### 2.4. RNA-Seq Analysis

Quality control and adaptor trimming were performed on the RNA sequence raw data FASTQ files using CLC Genomic Workbench software (20.0.4). The reference genome and annotations were downloaded from the Ensembl database. Data were mapped to the turkey genome (Meleagris Gallopavo) using CLC Genomic Workbench software (20.0.4). Differential expression analysis was performed using CLC Genomic Workbench to distinguish differentially expressed genes (DEGs) between the HFE and LFE groups. Finally, the DEGs were declared at a significant level of |(fold change)| > 2, raw *p*-value < 0.05, and FDR < 0.05.

### 2.5. Identification of Differently Expressed Genes (DEGs) and Function Annotation Analysis

Gene ontology (GO) terms and the Kyoto Encyclopedia of Genes and Genomes (KEGG) pathways were studied through the Database for Annotation, Visualization, and Integrated Discovery (DAVID) (version 6.8, https://david.ncifcrf.gov/, accessed on 1 August 2020) (*p*-value < 0.05). The Search Tool for the Retrieval of Interacting Genes (STRING) database was applied to construct the protein-protein interaction (PPI) network. Then, Cytoscape was used for visualizing the PPI network (http://cytoscape.org/, accessed on 1 August 2020). The CytoHubba application in Cytoscape was used to investigate the hub genes using four centrality methods, including maximal clique centrality (MCC), degree, maximum neighborhood component (MNC), and closeness.

The Molecular Complex Detection (MCODE) application in Cytoscape was applied to investigate the modules of the PPI network. The criteria settings of MCODE are as follows: degree cutoff = 2, node score cutoff = 0.2, k-core = 2, maximum depth = 100. Additionally, pathway and function enrichment analyses were conducted for genes in the modules using the ClueGO application of Cytoscape.

### 2.6. Gene Set Enrichment Analysis (GSEA)

All the expressed genes were used for GSEA analysis. Gene set analyses were performed using GSEA software (http://software.broadinstitute.org/gsea/index.jsp, accessed on 25 August 2020) based on the KEGG gene set collections (MSigDB v7.0, broad institute, Cambridge, MA, USA). This software sorted all the expressed genes based on the significance of differential gene expression between the two groups. A normalized enriched score (NES) was calculated for each gene set. The significant enrichment of the gene set was selected based on the absolute values of NES > 1, the nominal *p*-value of NES ≤ 0.05, and the false discovery rate (FDR) ≤ 0.25.

### 2.7. Quantitative RT-PCR Confirmations

A quantitative reverse transcription PCR (qRT-PCR) assay was used for confirming the differential expression results of 20 DEGs that were randomly selected. The primers were designed using Primer Premier Software (version 5) and synthesized by the Cinaclone Co. (Tehran, Iran). The total RNA was used for first-strand cDNA synthesis using a RevertAid First Strand cDNA Synthesis kit (Thermo Scientific, Waltham, MA, USA, #K1621) according to standard protocols. All qRT-PCR reactions were performed in triplicate via a LightCycler detection assay (Roche Diagnostics Corporation, Indianapolis, IND, USA) using SinaSYBERBlue HS-qPCRMix. The thermal cycle conditions were as follows: 1 cycle of pre-incubation at 94 °C for 3 min, 40 cycles of amplification (95 °C for 30 s, 59 °C for 30 s, and 72 °C for 30 s). The relative gene expressions of DEGs were calculated as

Fold change = 2^−ΔΔCt^ (2)

The primer sequences of all the genes including the target genes and three housekeeping genes are presented in Table 1. Selecting proper reference genes is one of the most important points in qPCR data analysis and plays a crucial role in the correct assessment of gene expression. Stable reference genes for the qPCR method were determined among three housekeeping genes (RPS7, 18 S, and GADPH) using the GeNorm algorithm [15].

## 3. Results

### Gene Expression Profile

**Figure 1:**
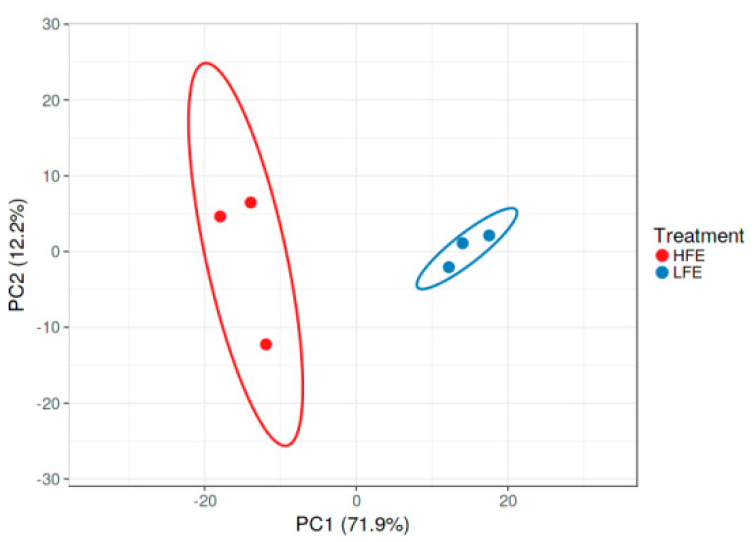
Classification of HFE and LFE turkeys according to the results of multiple corresponding analyses. The results show that HFE and LFE turkeys can be divided into two groups.

Breast muscle samples of HFE (*n* = 3) and LFE (*n* = 3) native turkeys were used for RNA-Seq analysis. The results of the quality control indicated that short reads were sequenced based on an appropriate form. Trimming was carried out according to the Phred score and the nucleotide contribution. An average of 78.5 million clean reads per sample were mapped back to the *Meleagris gallopavo* reference genome with a mean of 73.97% mapping efficiency. Detailed information on the data quality and mapping statistics is presented in Table 2. The classification of the HFE and LFE turkeys according to the results of PCA is presented in Figure 1. The figure shows that the HFE and LFE groups were very different from each other; HFE and LFE turkeys can be separated into two different groups.

**Figure 2:**
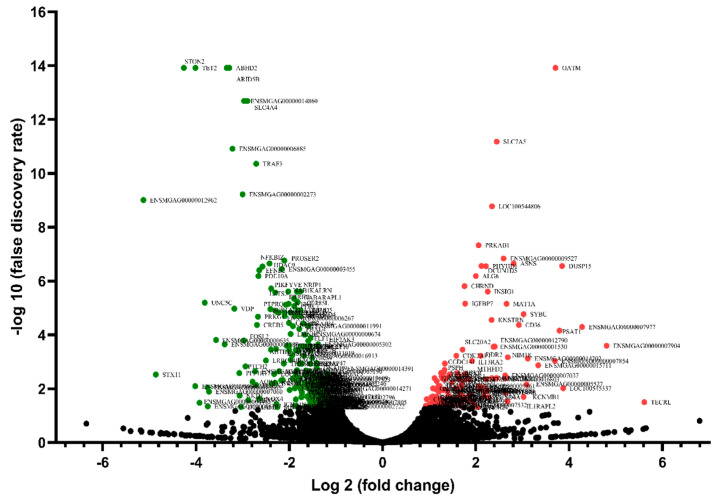
The volcano plot indicates the −log10 (*p*-value) for genome-wide genes (*y*-axis) plotted against their respective log2 (fold change) (*x*-axis). Red dots represent up-regulated genes and green dots represent down-regulated genes.

Differential gene expression analysis was applied to analyze the transcriptional variations between the HFE and LFE groups. In total, 365 significant DEGs (based on |(fold change)| > 2, *p*-value < 0.05, and FDR < 0.05) were found in response to divergent FE. Of these genes, 127 were up-regulated and 238 were down-regulated in the HFE group. A volcano plot reporting −log (false discovery rate) against log (fold change) is presented in Figure 2.

**Figure 3:**
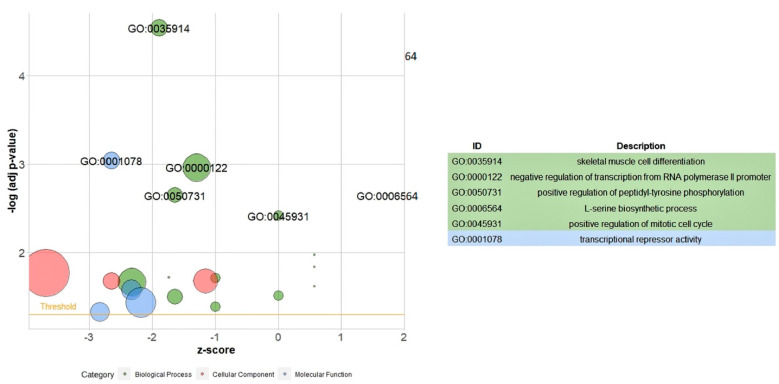
Gene ontology analysis of all DEGs. The bubble plot shows the GO enrichment of differentially expressed genes in five biological process terms and one molecular function.

An analysis of gene ontology (GO) enrichment was conducted for all DEGs (Figure 3). The bubble plot shows the GO enrichment of differentially expressed genes in five biological process terms including “skeletal muscle cell differentiation”, “negative regulation of transcription from RNA polymerase II promoter”, “positive regulation of peptidyl-tyrosine phosphorylation”, “L-serine biosynthetic process” and “positive regulation of mitotic cell cycle”. The GO analysis showed one term in molecular functions, “transcriptional repressor activity”.

To identify the potential functional classes of DEGs, a functional enrichment analysis including pathway enrichment and analysis of GO enrichment was conducted on the up-regulated and down-regulated DEGs separately. Out of 127 overexpressed genes, 96 genes were identified in pathway analysis. Major pathways including the “glycine, serine, and threonine metabolism”; “biosynthesis of amino acids”, “adipocytokine signaling pathway”, and “metabolic pathways” were statistically significant. In the pathway analysis of down-regulated DEGs, the major significant pathways were “dorso-ventral axis formation” and “regulation of actin cytoskeleton” (Table 3).

Moreover, the significantly enriched GO terms were analyzed in three terms: Biological process (BP), Cellular component (CC), and Molecular function (MF). The most important part of the ontology of up-regulated genes in the BP term was related to the “L-serine biosynthetic process” (GO: 0006564). The most significant ontology in CC term was related to “extracellular space” (GO: 0005615). In the MF term, the only significant ontology was related to “phosphomannomutase activity” (GO: 0004615) (Table 4).

The most significant part of the ontology of the down-regulated genes in the BP term was related to “skeletal muscle cell differentiation” (GO: 0035914). The most significant ontology in the CC term was related to “nucleus” (GO: 0005634). In the MF term, the most significant ontology was related to “transcriptional repressor activity, RNA polymerase II core promoter proximal region sequence-specific binding” (GO: 0001078) (Table 5).

For revealing the interaction information and for further analysis of the DEGs, the PPI network of the DEGs was investigated. This PPI network, created by the STRING database and visualized through Cytoscape software, included 301 nodes and 412 edges.

**Figure 4:**
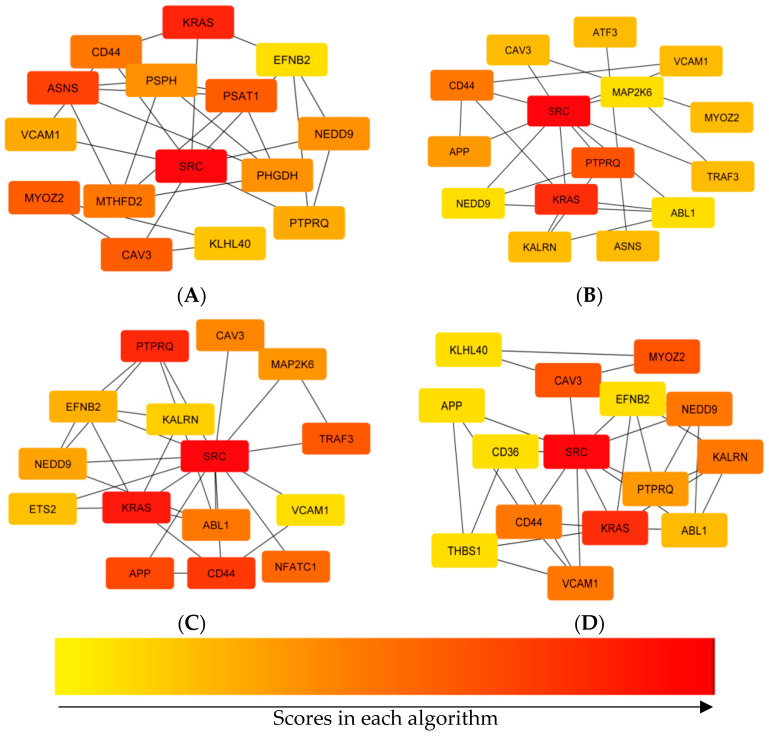
Comparing hub genes that were identified using four centrality algorithms of CytoHubba. (**A**) MCC, (**B**) degree, (**C**) closeness, (**D**) MNC.

**Figure 5:**
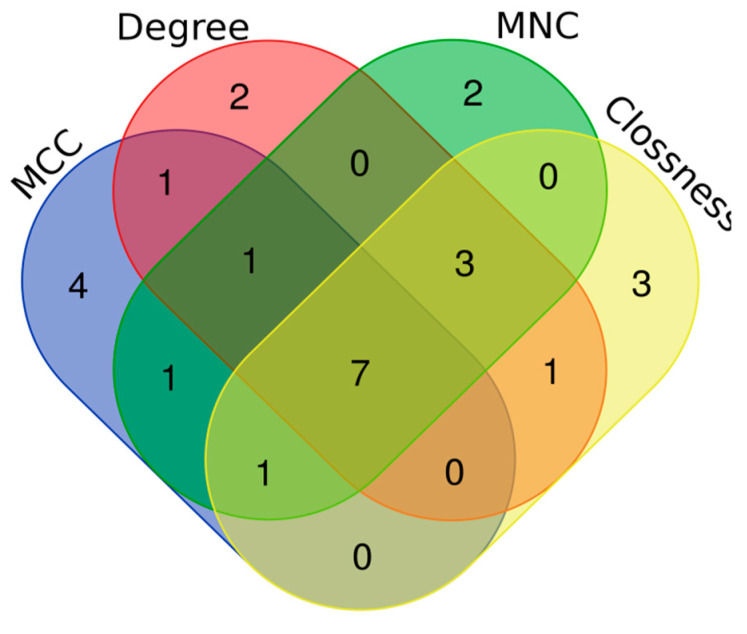
Venn plot to identify significant hub genes created by four centrality algorithms. Areas with different colors are related to different methods. The cross areas show the common DEGs. There are seven genes in concurrent areas.

Using four centrality algorithms of CytoHubba (MCC, MNC, degree, and closeness), 15 hub genes were evaluated and visualized. (Figure 4). The intersections of these four methods were calculated and seven hub genes, including *CD44*, *VCAM1*, *KRAS*, *NEDD9*, *SRC*, *PTPRQ*, and *CAV3* were identified (Figure 5).

**Figure 6:**
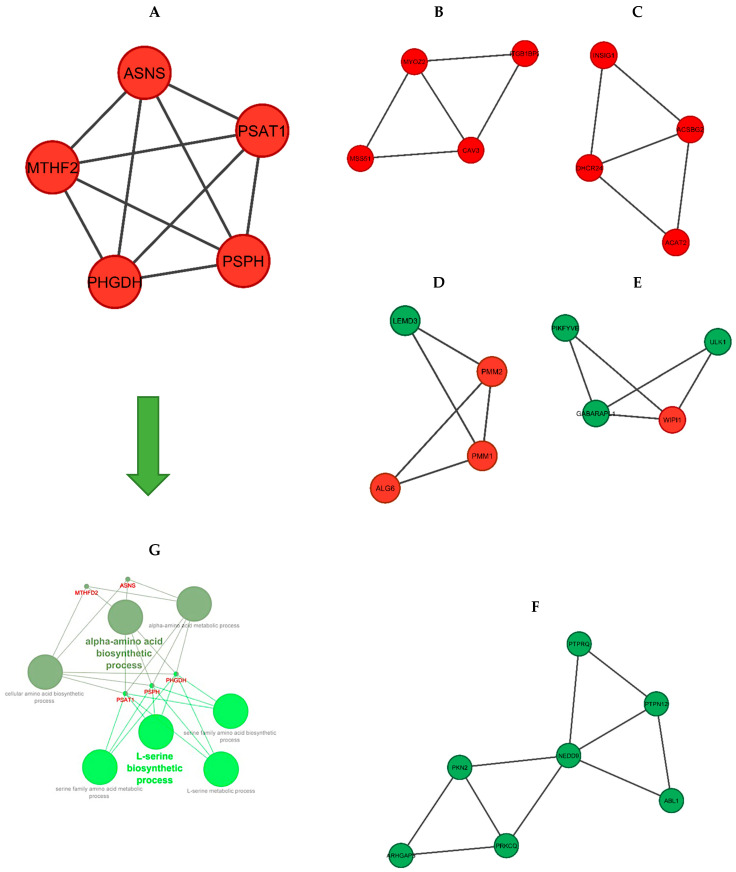
The significant modules of the DEGs PPI network. (**A**–**F**): Modules 1–6. (red nodes: up-regulated genes, green nodes: down-regulated genes). (**G**): Functional enrichment analysis of module 1. The two green spectra represent two different gene ontologies in which the genes of this module are involved (L-serine biosynthetic process and alpha-amino acid biosynthetic process).

The six significant modules with an MCODE score of higher than three were identified including module one (MCODE score = 5) and module two to module six (MCODE score = 3.333), which were created using MCODE (Figure 6). Then, biological functional enrichment analysis of the genes of each module was conducted using ClueGO. Only module one showed significant results. The most important biological processes of this module were the alpha-amino acid biosynthetic process and the L-serine biosynthetic process.

**Figure 7:**
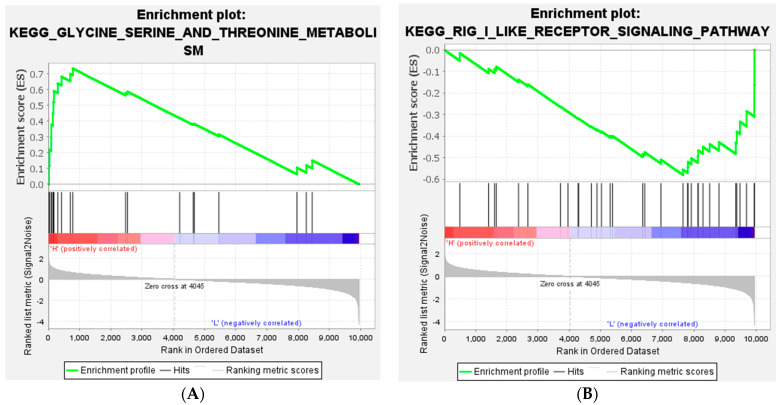
Gene set enrichment analysis (GSEA). The enrichment plot of most significant expressed in (**A**) HFE (glycine, serine, and threonine metabolism) and (**B**) LFE (RIG-I-like receptor signaling pathway) birds.

The total gene expression levels between the HFE and LFE groups were investigated by GSEA, which was performed using a KEGG-based list including 41 gene sets. Only 1 gene set was identified as significantly enriched in HFE, and 14 gene sets were enriched in LFE (FDR ≤ 0.25). Usually however, an FDR of less than 0.25 can be considered a significant gene set in GSEA results (Table 6). The enrichment plot of the most significantly expressed genes in HFE and LFE birds is presented in Figure 7.

**Figure 8:**
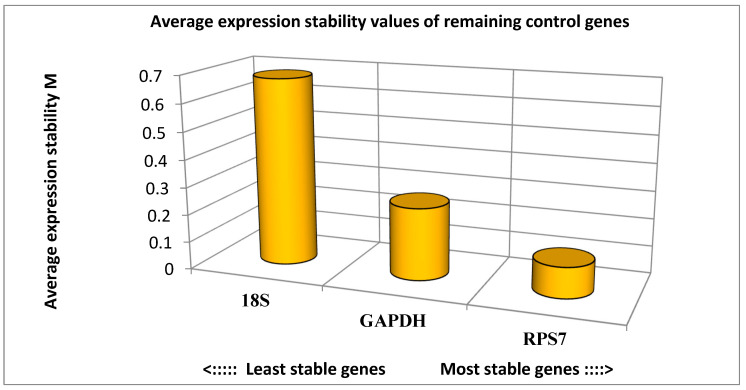
Expression stability analysis of reference genes in breast muscle tissue of turkeys. The most stably expressed genes have lower M values.

To validate the RNA-Seq expression results, twenty genes were randomly selected. A statistical analysis of gene expression stability was performed using the GeNorm algorithm (Figure 8). The results showed that the expression stability of the three reference genes from high to low was RPS7, GAPDH, and 18S. Therefore, RPS7 was used as a reference gene for qPCR experiments.

**Figure 9:**
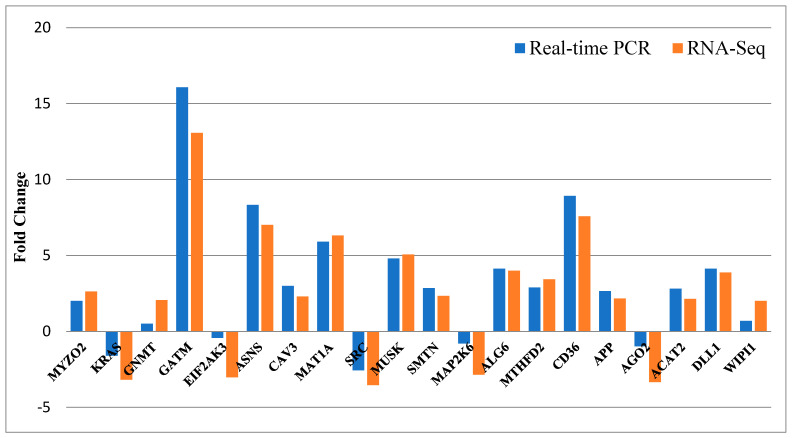
The results of qRT-PCR confirmation for 20 selected differentially expressed genes. The *X*-axis shows 20 selected genes for qRT-PCR assays, and the *Y*-axis demonstrates the log2 (fold change) derived from RNA-Seq and qRT-PCR.

The comparative outcomes of the fold changes predicted by RNA-Seq and qRT-PCR were represented in Figure 9. Approximately all of the selected genes showed concordant expression patterns between the RNA-Seq and qRT-PCR results.

**Figure 10:**
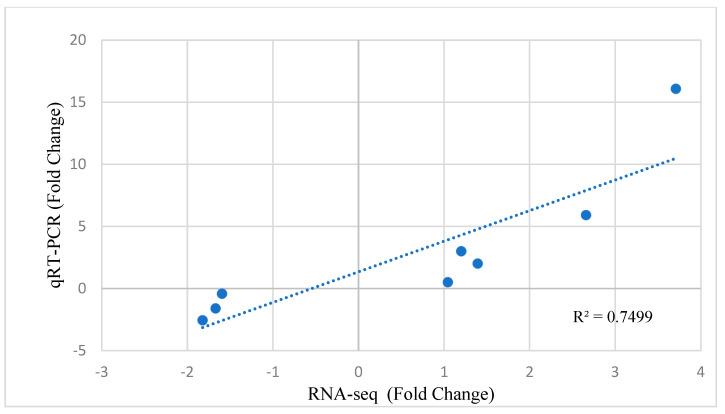
Quantitative real-time PCR (qRT-PCR) validation and linear regression analysis of RNA sequencing of selected DEGs.

The results of real-time PCR also showed a similar expression pattern to the analysis results from RNA sequencing. Therefore, there was a good agreement between the real-time PCR and RNA-Seq results (Figure 10).

## 4. Discussion

In this research, breast muscle transcriptome data were analyzed and DEGs obtained from native male turkeys individually phenotyped for feed efficiency using RNA-Seq technology. Functional annotation, investigation of the PPI network, and module analysis were conducted (see Methods). Following a key nodes analysis of the PPI network, the hub genes were found. In addition, a functional enrichment of all the expressed genes was performed between HFE and LFE turkeys.

A list of DEGs was generated using differential expression analysis which needed further analysis to create an overview of the differences between the two groups. In total, 365 significant DEGs (127 up-regulated and 238 down-regulated) were found in response to divergent FE.

An ontology annotation of all the DEGs revealed some GO terms indicating that the most significantly enriched GOs were related to skeletal muscle cell differentiation, negative regulation of transcription from the RNA polymerase II promoter, positive regulation of peptidyl-tyrosine phosphorylation, the L-serine biosynthetic process, and positive regulation of the mitotic cell cycle in the BP term; and transcriptional repressor activity in the MF term. Therefore, most GO terms of all the DEGs were enriched in the metabolic process and cell differentiation, both of which are important in growth and feed efficiency traits. A pathway enrichment was conducted on the up-regulated DEGs. Major pathways including “glycine, serine, and threonine metabolism”, “biosynthesis of amino acids”, “adipocytokine signaling pathway”, and “metabolic pathways” were statistically significant.

Kong et al. (2011) reported that there was a down-regulation of cytoskeletal organization genes in the HFE phenotype [5]. The glycine, serine, and threonine metabolism pathway is one of the metabolic pathways that was significant in the duodenum and adipose tissue in cattle with divergent feed efficiency on several tissues [16]. The glycine, serine, and threonine metabolism pathway was reported to be one of the significant metabolic pathways in Holstein and Jersey dairy cows with high and low feed efficiencies [17]. Yang et al. (2017) reported that the glycine, serine, and threonine metabolism pathway had higher abundances in HFE pigs [18].

Glucose is the main source of metabolic energy in the body. When glucose enters the cell, glycolysis starts in the cytoplasm. The glycolysis pathway provides an intermediate metabolite, 3-phosphoglycerate, which is catalyzed into serine by *PHGDH*, *PSAT1*, and *PSPH* [19]. Then, serine can be converted into glycine. Additionally, glycine can be converted to threonine. This pathway provides precursors for purine biosynthesis and the tricarboxylic acid (TCA) cycle [20]. The TCA cycle generates ATP for protein production. It has been reported that HFE pigs accumulate more muscle mass in comparison to LFE pigs in their breast muscle [21]. Thus, the results of the present study in turkeys suggest that the HFE animals may consume more glucose in the muscle compared to LFE birds to produce ATP for protein deposition.

**Figure 11:**
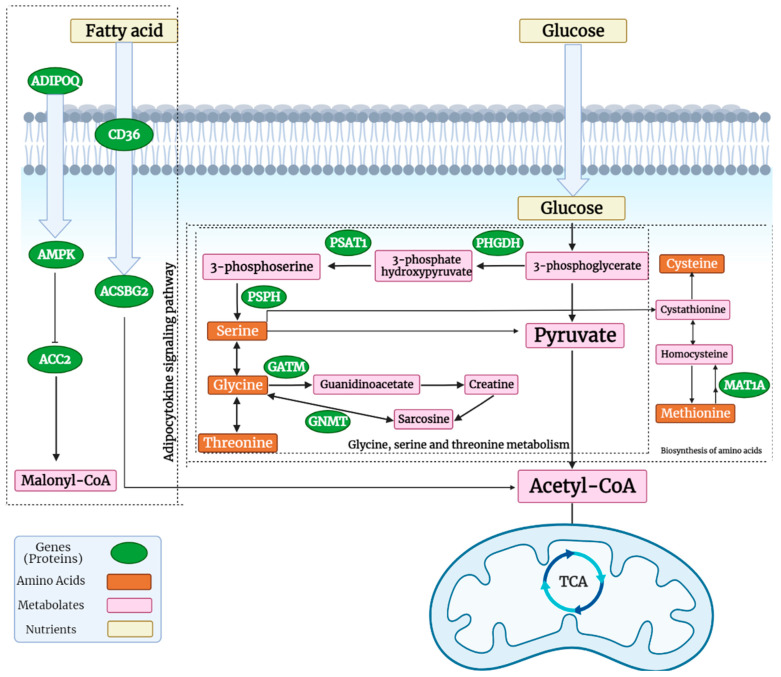
Three important pathways which were significant in HFE turkeys, including glycine, serine, and threonine metabolism, the adipocytokine signaling pathway, and the biosynthesis of amino acids (created with BioRender.com, accessed on 10 April 2022).

On the other hand, glycine can be converted to creatine by *GATM* which was over-expressed (or up-regulated) in the HFE turkey phenotype. Creatine has some anti-inflammatory and anti-oxidant characteristics, increases insulin sensitivity, and creatine metabolism and *GATM* has an important role in energy expenditure and defense against diet-induced obesity [22]. The entirety of this process is more active in HFE birds. Therefore, HFE turkeys may have better glucose absorption and have more active glycolysis or gluconeogenesis pathways. Additionally, the biosynthesis of amino acids provides a situation for producing more protein. Therefore, increasing this pathway in the breast muscle of birds results in heavier breast muscles in HFE turkeys (Figure 11).

Fonseca et al. (2019) found metabolic pathways related to feed efficiency in beef cattle using liver proteomics. They revealed that the biosynthesis of amino acids is one of the main pathways associated with FE [23].

The adipocytokine signaling pathway was another of the significant pathways. Adiponectin production (a protein encoded by the *ADIPOQ* gene) is negatively associated with increasing the volume and number of adipocytes. In circulation, adiponectin increases the transport of plasma fatty acids and glucose into cells. It is involved in the control of fat metabolism and has anti-inflammatory activities. Adiponectin also induces AMPK activation, which stimulates skeletal muscle glucose uptake and fatty acid oxidation. It also induces gluconeogenesis through the inhibition of the PEPCK and G6PC proteins. Therefore, these processes in HFE turkeys result in better glucose absorption and fat metabolism. The fatty acid translocase gene *CD36* is a membrane receptor that facilitates the uptake of long-chain fatty acids. It is also involved in the adipocytokine signaling pathway. Finally, it provides precursors of acetyl-CoA for the TCA cycle. Therefore, a better uptake of fatty acids, stimulating gluconeogenesis and increasing insulin sensitivity, occurs through the adipocytokine signaling pathway in HFE turkeys [24]. The results of a study on Holstein cows showed that the adipocytokine signaling pathway is one of the significant pathways in Holstein cows with high and low feed efficiencies [25]. In addition, Lassiter et al. (2006) reported an increased expression of AMPK in the HFE phenotype of pedigree male broilers [26].

A pathway analysis of the down-regulated DEGs in the current study showed that the major significant pathways were “dorso-ventral axis formation” and “regulation of actin cytoskeleton”. Moreover, Yang et al. (2020) found that one of the significant modules in which the DEGs of native chickens were enriched was the regulation of the actin cytoskeleton that is involved in the regulation of cell motility [14].

Apparent metabolizable energy is a method to assess energy utilization for feed efficiency. Pezeshkian et al. (2020) investigated the apparent metabolizable energy of chickens and showed that down-regulated genes were enriched in some pathways including regulation of the actin cytoskeleton [27].

The ontology annotation of the DEGs revealed several biological events mostly associated with the biosynthesis of amino acids process and growth. They include the L-serine biosynthetic process, the GDP-mannose biosynthetic process, skeletal muscle tissue growth, amyloid fibril formation, the regulation of cardiac muscle contraction by regulation of the release of sequestered calcium ions, and the negative regulation of heart rate. “Low-density lipoprotein particle” refers to a process in which a low-density lipoprotein particle is removed from the blood. “Protein homotetramerization” is the process of forming protein homotetramers, which consist of four identical subunits of proteins. Protein and lipid metabolisms are important factors in feed efficiency [28]. Therefore, the higher expression of genes of these processes in HFE-phenotyped male native turkeys can be interpreted.

Some genes were involved in multiple pathways and GO terms. Among the DEGs, *GATM* was the most highly expressed gene. The glycine aminotransferase (*GATM*) gene is involved in glycine, serine, and threonine metabolism and other metabolic pathways. This enzyme converts glycine to arginine by guanidino acetate. Moreover, it was involved in the gene set of glycine, serine, and threonine metabolism in GSEA analysis. This gene was reported as a gene with different expression in a study that investigated the molecular basis of feed efficiency in broilers [29]. Mckenna (2018) investigated the molecular control of feed efficiency in beef cattle, and the *GATM* gene was introduced as a gene with different expression in muscle tissue between animals with a high and low feed efficiency [30].

Phosphoglycerate dehydrogenase (*PHGDH*), phosphoserine aminotransferase 1 (*PSAT1*), and phosphoserine phosphatase (*PSPH*) are genes in the serine biosynthesis process [31]. Fonseca et al. (2019) performed liver proteomics which elucidated the metabolic pathways associated with feed efficiency in beef cattle, and the *PSAT1* gene was identified as a DEG involved in different metabolic pathways [23]. The *PSPH* gene is known as a DEG between cows with high and low residual feed intake [32]. Keogh et al. (2016) showed that the *PSPH* gene was up-regulated in HFE animals and was therefore introduced as a marker for feed efficiency [33].

To further evaluate the intrinsic relationship between DEGs, mapping of the PPI networks was performed. Analyzing PPI networks is essential for understanding the molecular basis for complex traits. In the current study, after construction of the PPI network, the top centrality hub genes were achieved through four centrality algorithms. Finally, seven hub genes, including *CD44*, *VCAM1*, *KRAS*, *NEDD9*, *SRC*, *PTPRQ*, and *CAV3* were identified.

During inflammation, vascular cell adhesion molecule 1 (*VCAM1*) is mainly expressed in endothelial cells and causes vascular inflammation [34]. *VCAM1* was reported as a key gene related to the feed efficiency of native chickens [14]. *KRAS* plays an important role in regulating cell proliferation and was down-regulated in HFE turkeys in the current study. In a RNA-Seq transcriptomics and pathway analysis study on dairy cattle with a high and low residual feed intake, one of the genes with a significantly different expression was *KRAS* gene [35]. Zarek et al. (2017) investigated gene expression related to weight gain and feed intake in liver tissue and showed that the *KRAS* gene was one of the DEGs between groups with a different phenotype of weight gain and feed intake [36]. Mukherjee et al. (2020) found that the phosphatidylinositol phosphatase (*PTPRQ*) gene was up-regulated in low growth bovines. This gene can regulate cell survival and proliferation [37]. The caveolin 3 gene (*CAV3*) is a member of the caveolin family and acts as a specific muscle isoform of the caveolin protein. Lassiter et al. (2019) showed that the *CAV3* and *MYOZ2* genes were among the DEGs that were involved in muscle development and differentiation in broilers with a high and low feed efficiency [38]. The *SRC* gene encodes protein tyrosine kinases. Lam et al. (2021) used RNA-Seq to identify that the *SRC* gene is among the most important genes with different expression in a group of animals with low feed efficiency [39]. Bottje et al. (2017) investigated broilers with divergent feed efficiency and one of the top enrichment down-regulated genes in the HFE group was *CD44* [40]. Onteru et al. (2013) found that *NEDD9* (neural precursor cell expressed, developmentally down-regulated 9) was one of the significant single nucleotide polymorphisms between pigs with a low and high residual feed intake (RFI) [41].

To further analyze the PPI network, we investigated the significant modules and found six modules with an MCODE score of higher than three. Both KEGG pathway and GO analyses were performed using the ClueGO plugin, and only the first module (including *PSPH*, *PHGDH*, *PSAT1*, *MTHFD2,* and *ASNS*) showed significant enrichment terms. The most important of these enrichment analyses were the alpha-amino acid biosynthetic process and the L-serine biosynthetic process. As mentioned before, the amino acid and protein biosynthetic process has a significant effect on the feed efficiency of animals.

In the current study, we applied the GSEA method to change the RNA-Seq data into biological interpretations. Therefore, there are not any limitations and thresholds for the expressed genes to perform function analysis. In addition, GSEA can reliably identify gene sets with biological significance [42]. As a result, the most significant gene set that was highly expressed in HFE was glycine, serine, and threonine metabolism. As mentioned before, amino acid metabolism has a key effect on feed efficiency. On the other hand, the most significant gene lists in the LFE group were the RIG-I-like receptor signaling pathway and the Jak-STAT signaling pathway. Both of these pathways are related to the inflammatory response and the immune response. This might suggest increased oxidative stress in the LFE phenotype which agrees with findings in the muscle [43], intestines [44], liver [45], and heart [46] and those of the study of Lassiter et al. (2006) on high and low FE broilers [24]. It has been reported that the high production of pro-inflammatory agents may cause damage to the intestinal integrity and decrease feed efficiency [47]. These results showed that pathways associated with the inflammatory and immune responses are related to feed efficiency. The study of Yang et al. (2020) reported an overexpression of genes related to the Jak-STAT signaling pathway in native chickens with a high RFI [14].

To confirm the putative results from RNA-Seq, we randomly selected a subset of DEGs for qRT-PCR assays. Overall, there was an excellent agreement and high concordance between the computational and experimental results, which revealed a good detection sensitivity and accuracy.

## 5. Conclusions

This study provided new insights into the differences in the transcriptomes of breast muscle tissue between high and low feed efficiency native male turkeys. In the pathway analysis of up-regulated genes, major pathways included the “metabolism of glycine, serine, and threonine”; the “adipocytokine signaling pathway” and the “biosynthesis of amino acids”. In the pathway analysis of down-regulated genes, the major pathways included “dorso-ventral axis formation” and “actin cytoskeleton regulation”. Our results showed that feed efficiency is associated with metabolic pathways, especially with the metabolism of various amino acids and protein deposition. Furthermore, functional analysis and protein network interaction analysis revealed that genes including *GATM*, *PSAT1*, *PSPH*, *PHGDH*, *VCAM1*, *CD44*, *KRAS*, *SRC*, *CAV3*, *NEDD9,* and *PTPRQ* were key genes for feed efficiency. The key genes introduced in the current study can be used as biomarkers to perform breeding work in the native turkey population of Iran. In addition, this study is the first study to examine transcripts related to feed efficiency in turkey species. The results of this study can be compared with the results of other poultry such as broilers and can be used to explain the comprehensive atlas of genes involved in poultry feed efficiency.

## Figures and Tables

**Table 1:** The primer sequences of target genes and housekeeping genes used in this study.

Gene Name	Accession Number		Sequence of Primers (5′ → 3′) *	Product Length (bp)
GATM	XM_019619468	Forward	CGTTTAATATCATTGGACCTGG	216
		Reverse	TTGAAGTCTCATTGGCATCG	
GNMT	XM_010706618	Forward	CTGGAGCAGGACCTGGAGAAG	246
		Reverse	CTTGGTCAGGTCGCTCTTGTAG	
KRAS	NM_001303223	Forward	CTGAAGATGTCCCAATGGTGCT	182
		Reverse	GTGTTTTCTGATTCTCGAACTAATG	
MAT1A	XM_003207784	Forward	ATGCCAAAGTTGCTTGTGAGAC	192
		Reverse	GTGATTGCTGTTCCAGTGCCA	
CAV3	XM_003210208	Forward	CAAAGCGGATCAACGAAGAC	200
		Reverse	GATGAGGGCGAAGAGGAAGC	
MYOZ2	XM_003205713	Forward	CACAACGAAACAGTACGCAAGG	196
		Reverse	TGGGTGAGACTCAATACAATGAG	
MUSK	XM_010726142	Forward	GAATATAACCAGGACTTGCTACAG	164
		Reverse	CAGTAATTCTCAGCATCAGACAG	
SMTN	XM_010720500	Forward	GACAGGCAGCATCTTTGACC	135
		Reverse	CTGTGAGGTTGATGTCTTGGG	
MAP2K6	XM_010721466	Forward	TTTAGCAACCGAGTCAACGA	105
		Reverse	TTTAGCAACCGAGTCAACGA	
ALG6	XM_003208901	Forward	CAAGAAGGGACTGAAAGGAAAGG	169
		Reverse	CTACTTTATCCTCAAACAAGCCTC	
MTHFD2	XM_003206271	Forward	TCATAAAGAGAACAGGCATCCCA	154
		Reverse	TAACGGTGTGATATTGTGACTGTG	
CD36	XM_010726962	Forward	CCAGGAAGCTCTGTTTACAGG	121
		Reverse	TATCGCACCCTATATGTGTAAGGT	
APP	XM_010722443	Forward	GAAGTTGTCAGAGTCCCTACC	173
		Reverse	TTCTGCCTCCTCCCATTCTC	
AGO2	XM_031552935	Forward	TGGAAGAGATAAAGTGGAGTGGA	156
		Reverse	CTGGATGGTTTCAAATGGGAC	
ACAT2	XM_003204090	Forward	AGGAAAGCTATTGACAAAGCCA	186
		Reverse	CAAGGATGCGACAACCAGAG	
DLL1	XM_031552428	Forward	CTTGTGCTAATGGAGCCCAG	196
		Reverse	GCAGTTCTTCCCGTTGTATCC	
WIPI1	XM_010721504	Forward	CAGGTTATTCGGAGGATGGT	118
		Reverse	ACGGCACAAGATTATAGGAGGA	
ASNS	XM_019616348	Forward	ATATTTCCATAAGGCACCATCTCC	159
		Reverse	GTAAGAAGTAAGCGATGATCCAG	
SRC	XM_003211956	Forward	CAGCAAGAGCAAACCCAAAGA	103
		Reverse	CTTGTTGGGGGTCTGCGAG	
EIF2AK3	XM_010710584	Forward	GAGTCAAGACCCTGAGCGATGT	178
		Reverse	GGTTTGGCTGGGAGTTCCA	
18S	AJ419877	Forward	CTGCCCTATCAACTTTCGATGG	171
		Reverse	GGATGTGGTAGCCGTTTCTCA	
GAPDH	NM_001303179	Forward	CCCAGAACATCATCCCAGCAT	137
		Reverse	ACGGCAGGTCAGGTCAACAAC	
RPS7	NM_001285787	Forward	TGAAGTAGGTGGTGGCAGGAA	165
		Reverse	CTCGTTGGCTTGGGCAGAA	

* All primers were designed specifically for the current study.

**Table 2:** A summary of short reads before and after trimming and mapped reads which were aligned against the reference genome for HFE and LFE turkeys.

Sample	Raw Reads	Trimmed Reads	Mapped Ratio (%)
H1	71,407,390	71,397,082	74.56
H2	81,000,062	80,986,086	72.76
H3	80,467,686	80,451,478	74.25
L1	93,253,472	93,235,602	74.56
L2	75,960,342	75,946,338	73.1
L3	69,196,190	69,186,026	74.63

**Table 3:** Pathway analysis of DEGs in HFE compared to LFE turkeys (genes with red font color represent up-regulated genes, and genes with green font color represent down-regulated genes).

Term	Biological Process	Count	*p*-Value	Genes
Up-regulated				
mgp00260	Glycine, serine, and threonine metabolism	6	1.64 × 10^−5^	*GATM* , *PHGDH*, *GNMT*, *PSPH*, *PSAT1*, *LOC100550886*
mgp01230	Biosynthesis of amino acids	5	0.001	*MAT1A* , *PHGDH*, *PSPH*, *PSAT1*, *LOC100550886*
mgp04920	Adipocytokine signaling pathway	5	0.0022	*CD36* , *ACSBG2*, *AMPK*, *ACC2*, *ADIPOQ*
mgp01100	Metabolic pathways	17	0.021	*POLR3H* , *GATM*, *ACSBG2*, *ASNS*, *ALG6*, *ACC2*, *PSPH*, *ACAT2*, *PMM2*, *PMM1*, *MTHFD2*, *MAT1A*, *PHGDH*, *PSAT1*, *DCXR*, *DHCR24*, *LOC100550886*
Down-regulated				
mgp04320	Dorso-ventral axis formation	4	0.002	*KRAS* , *CPEB3*, *ETS2*, *CPEB4*
mgp04810	Regulation of actin cytoskeleton	7	0.022	*ENAH* , *PIKFYVE*, *MRAS*, *SRC*, *MSN*, *KRAS*, *NCKAP1L*

**Table 4:** Ontology analysis of up-regulated genes in HFE compared to LFE turkeys.

Term	Biological Process	Count	*p*-Value	Genes
GO:0006564	L-serine biosynthetic process	3	3.09 × 10^−4^	*PHGDH*, *PSPH*, *PSAT1*
GO:0009298	GDP-mannose biosynthetic process	2	0.03	*PMM2*, *PMM1*
GO:0048630	skeletal muscle tissue growth	2	0.03	*DLL1*, *CHRND*
GO:1990000	amyloid fibril formation	2	0.03	*APP*, *CD36*
GO:0034383	low-density lipoprotein particle clearance	2	0.03	*CD36*, *ADIPOQ*
GO:0010881	regulation of cardiac muscle contraction by regulation of the release of sequestered calcium ion	2	0.03	*PLN*, *FKBP1B*
GO:0051289	protein homotetramerization	3	0.033	*GNMT*, *ACC2*, *DCXR*
GO:0010459	negative regulation of heart rate	2	0.04	*PLN*, *FKBP1B*
Cellular component				
GO:0005615	extracellular space	9	0.033	*CPNE9*, *MTHFD2*, *APP*, *CD36*, *IGFBP7*, *ANGPTL1*, *SPON2*, *ADIPOQ*, *ANGPT4*
GO:0033017	sarcoplasmic reticulum membrane	2	0.039	*PLN*, *FKBP1B*
GO:0070062	extracellular exosome	21	0.046	*CPNE9*, *SPON2*, *APP*, *SLC20A2*, *DCXR*, *PMM2*, *ADIPOQ*, *ACOT11*, *SEMA3G*, *SLC1A4*, *ACAT2*, *MYLK*, *GATM*, *TP53I3*, *BAIAP2L1*, *LOC100551072*, *PSAT1*, *PHGDH*, *IGFBP7*, *ANGPTL1*, *DDR2*
GO:0030018	Z disc	3	0.0498	*ITGB1BP2*, *MYOZ2*, *FKBP1B*
Molecular function				
GO:0004615	phosphomannomutase activity	2	0.02	*PMM2*, *PMM1*

**Table 5:** Ontology analysis of down-regulated genes in HFE compared to LFE turkeys.

Term	Biological Process	Count	*p*-Value	Genes
GO:0035914	skeletal muscle cell differentiation	6	4.41× 10^−5^	*BTG2*, *MYF6*, *HIVEP3*, *FOXN2*, *ATF3*, *BCL9L*
GO:0050731	positive regulation of peptidyl-tyrosine phosphorylation	5	0.003	*CD74*, *SRC*, *ABL1*, *ENPP2*, *RICTOR*
GO:0060213	positive regulation of nuclear-transcribed mRNA poly(A) tail shortening	3	0.0083	*BTG2*, *AGO2*, *CPEB3*
GO:0000122	negative regulation of transcription from RNA polymerase II promoter	10	0.0086	*PLK3*, *ZFHX3*, *MYOCD*, *NR4A3*, *TRPS1*, *NRIP1*, *OTUD7B*, *CPEB3*, *HDAC9*, *BARX2*
GO:0045944	positive regulation of transcription from RNA polymerase II promoter	12	0.0106	*RPS6KA3*, *MLLT10*, *SBNO2*, *ATXN7*, *NRIP1*, *AGO2*, *TET2*, *NFATC1*, *ETS2*, *BCL9L*, *ARNTL*, *FOSL2*
GO:0048536	spleen development	3	0.0252	*TET2*, *ABL1*, *JARID2*
GO:0070374	positive regulation of ERK1 and ERK2 cascade	5	0.0273	*CD74*, *SRC*, *ABL1*, *RAPGEF2*, *NOX4*
GO:0048008	platelet-derived growth factor receptor signaling pathway	3	0.0287	*CSRNP1*, *ZFAND5*, *ARID5B*
GO:0009791	post-embryonic development	4	0.03	*CSRNP1*, *AGO2*, *TET2*, *ARID5B*
GO:0030968	endoplasmic reticulum unfolded protein response	3	0.04	*STC2*, *EIF2AK3*, *ATF3*
GO:0048705	skeletal system morphogenesis	3	0.049	*CSRNP1*, *ZFAND5*, *ARID5B*
Term	Cellular Component			
GO:0005634	Nucleus	31	0.001	*CSRNP1*, *CCNJ*, *SRC*, *NAB1*, *CELF2*, *NEDD9*, *TNFAIP3*, *OTUD7B*, *ABHD5*, *UACA*, *BACH1*, *BARX2*, *NR3C2*, *NFIL3*, *SERTAD2*, *NFKBIZ*, *HIVEP3*, *SKIL*, *MYOCD*, *ARID5B*, *MSN*, *BTBD7*, *FOXN2*, *PISD*, *NR4A3*, *ALOX5AP*, *AGO2*, *MYF6*, *NOX4*, *CPEB3*, *CPEB4*
GO:0005667	transcription factor complex	7	0.0025	*ZFHX3*, *NR4A3*, *HDAC9*, *SKIL*, *BARX2*, *ETS2*, *ARNTL*
GO:0005730	Nucleolus	13	0.013	*PLK3*, *ZFHX3*, *DDX24*, *NUP153*, *STON2*, *PPM1E*, *BTBD10*, *NOL6*, *NRIP1*, *ABL1*, *KDM7A*, *ATF3*, *BCL9L*
Term	Molecular function			
GO:0001078	transcriptional repressor activity, RNA polymerase II core promoter proximal region sequence-specific binding	7	9.69E-05	*NFIL3*, *AEBP2*, *ZNF536*, *BACH1*, *SKIL*, *ATF3*, *ETS2*
GO:0043565	sequence-specific DNA binding	8	0.0059	*CSRNP1*, *ZFHX3*, *TRPS1*, *FOXN2*, *SKIL*, *ETS2*, *CREB5*, *NR3C2*
GO:0000978	RNA polymerase II core promoter proximal region sequence-specific DNA binding	8	0.009	*ELF1*, *NR4A3*, *AEBP2*, *IRF1*, *ZNF536*, *SKIL*, *ATF3*, *ETS2*
GO:0003713	transcription coactivator activity	6	0.01	*MYOCD*, *MAML1*, *SERTAD2*, *JMY*, *NRIP1*, *ARID5B*
GO:0008270	zinc ion binding	18	0.02	*ZFHX3*, *ANKIB1*, *ZFAND5*, *TET2*, *TNFAIP3*, *OTUD7B*, *NUP153*, *NR3C2*, *MLLT10*, *PIKFYVE*, *NR4A3*, *TRAF3*, *TRPS1*, *DHX58*, *ENPP2*, *HIVEP3*, *LONRF2*, *KDM7A*
GO:0046872	metal ion binding	13	0.028	*ZBTB21*, *ZBTB44*, *PPM1E*, *HDAC9*, *ZC3H12C*, *PDE10A*, *PDP2*, *AEBP2*, *MB*, *ZNF536*, *PRKCQ*, *ZNF644*, *PDE7B*
GO:0003700	transcription factor activity, sequence-specific DNA binding	7	0.035	*TRPS1*, *NFATC1*, *FOXN2*, *CREB5*, *ARNTL*, *FOSL2*, *NR3C2*

**Table 6:** List of all gene sets with an FDR ≤ 0.25.

KEGG Set	SIZE	NES	*p*-Value	FDR Q-Value	Higher Expression
KEGG_GLYCINE_SERINE_AND_THREONINE_METABOLISM	20	1.66	0	0.194	HFE
KEGG_RIG_I_LIKE_RECEPTOR_SIGNALING_PATHWAY	33	−1.85395	0	0.046	LFE
KEGG_JAK_STAT_SIGNALING_PATHWAY	70	−1.51451	0	0.188488	LFE
KEGG_DORSO_VENTRAL_AXIS_FORMATION	15	−1.46771	0	0.19519	LFE
KEGG_NOD_LIKE_RECEPTOR_SIGNALING_PATHWAY	30	−1.42309	0	0.200888	LFE
KEGG_WNT_SIGNALING_PATHWAY	96	−1.4685	0	0.202649	LFE
KEGG_NATURAL_KILLER_CELL_MEDIATED_CYTOTOXICITY	54	−1.43289	0	0.205478	LFE
KEGG_NEUROTROPHIN_SIGNALING_PATHWAY	92	−1.4886	0	0.205977	LFE
KEGG_PROGESTERONE_MEDIATED_OOCYTE_MATURATION	57	−1.48398	0	0.206473	LFE
KEGG_FC_EPSILON_RI_SIGNALING_PATHWAY	49	−1.49622	0	0.210286	LFE
KEGG_MAPK_SIGNALING_PATHWAY	163	−1.51628	0	0.212236	LFE
KEGG_HEDGEHOG_SIGNALING_PATHWAY	33	−1.44312	0	0.215766	LFE
KEGG_TOLL_LIKE_RECEPTOR_SIGNALING_PATHWAY	51	−1.52378	0	0.238885	LFE
KEGG_B_CELL_RECEPTOR_SIGNALING_PATHWAY	48	−1.51919	0	0.241404	LFE
KEGG_GNRH_SIGNALING_PATHWAY	61	−1.38625	0	0.246314	LFE

## Data Availability

Not applicable.
